# Species Diversity and Geographical Distribution of Poultry Ascaridids: A Scoping Review

**DOI:** 10.1155/japr/2396097

**Published:** 2026-05-15

**Authors:** Chet Raj Pathak, Deb Prasad Pandey, Prabhat Khanal

**Affiliations:** ^1^ Department of Veterinary Microbiology and Parasitology, Faculty of Animal Science, Veterinary Science and Fisheries, Agriculture and Forestry University, Rampur, Chitwan, Nepal, afu.edu.np; ^2^ Faculty Biosciences and Aquaculture (FBA), Nord University, Steinkjer, Norway, nord.no

**Keywords:** *Ascaridia* spp, distributions, diversity, *Heterakis* spp, nematodes

## Abstract

Poultry nematodes, particularly Ascaridid species, significantly affect avian health and productivity and are widely distributed globally. Yet despite over 80% of poultry in developing countries being raised under extensive rearing systems, knowledge on infection risks posed by different poultry nematodes remains limited. This scoping review synthesizes the knowledge based on the evaluation of existing literature, consolidating insights into the diverse Ascaridid species infecting poultry and their distribution, thereby contributing to the broader comprehension and management of avian nematodes. We found a total of 11 nematode species infecting 8 host types having median prevalence of 24% (IQR: 41.07%–12%). *Ascaridia galli* and *Heterakis gallinarum* were the cosmopolitan nematodes reported with median prevalences of 28.75% and 17.20%, respectively. These nematodes almost have a cosmopolitan distribution with a potentiality of transmission between domesticated and across wildlife‐poultry pathways. However, during this scoping review, analysis verifies that six *Ascaridia* and five *Heterakis* species are present in chickens worldwide, with Africa and Asia showing a higher prevalence of infection because of adopting long‐standing free‐range poultry farming. The occurrence and diversity of Ascaridid infections in poultry depend on geography, host type, diagnostic methods, and rearing practices, highlighting the need for effective disease management, biosecurity, and control of anthelmintic resistance.

## 1. Introduction

Poultry, a diverse group of birds, includes indigenous and commercial chicken varieties, Muscovy ducks, mallard ducks, turkeys, guinea fowl, geese, quail, pigeons, ostriches, and pheasants. They are raised in large and small operations, mainly for eggs, meat, and feathers worldwide [1]. Among these bird groups, chickens are poultry raised predominantly worldwide, ducks in Asia, turkeys in North America, and guinea fowl and geese in Africa and Asia [1]. The United Nations Food and Agriculture Organization (FAO) estimates the global poultry population to be around 27.9 billion heads, with chickens accounting for some 94% of the world’s poultry population, followed by ducks (3%) and turkeys (1%) [1]. Many of these chicken populations are kept in traditional free‐range poultry with single or multiple parasitic infections of different classes including Protozoa, Cestoda, Trematoda, and Nematoda either single or mixed [2–4]. Nematodes are the most critical gastrointestinal parasites of poultry because of their enormous number of species and widespread geographical distribution [2, 5–7]. Some of these nematodes are also reported in wild birds [8]. Therefore, listing all known nematodes from poultry and wild bird populations is essential to highlight the common species affecting poultry and wild birds.

The order Ascaridida is composed of Ascaridoidea and Dioctophymatoidea superfamilies. The Ascaridoidea is composed of Ascarididae and Anisakidae families [9]. The Ascarididae includes 11 genera, including *Ascaridia* and *Heterakis* [9]. Herein, the term “Ascaridids” represents Ascarididae. The genus *Ascaridia* consists of 41 species infecting domestic and wild birds worldwide [9–15], and *Heterakis* consists of 29 species infecting a diverse group of birds globally [9, 11, 16–18]. Because of the advancement of molecular biology, new species are added to the science periodically [19–22]. *Ascaridia* spp. inhabit the gastrointestinal tract, mainly the large intestine of domesticated, captive, and wild bird species [16–18], and *Heterakis* spp. inhabit the ceca of chickens and turkeys [23]. Both *Ascaridia* spp. and *Heterakis* spp. have a monoxenic direct life cycle involving embryonated eggs passed in feces, leading to larval development in the environment and eventual ingestion by birds [24]. In addition, sometimes earthworm other arthropods may act as a paratenic host for *Heterakis* spp. [25]. Therefore, it is necessary to update the species checklist of nematodes infecting poultry to understand the possible association of Ascaridid species diversity with the corresponding diversity of poultry hosts and biological traits [26].

The study of poultry nematodes, particularly Ascaridids, is a critical domain in veterinary parasitology due to its significant impact on avian health, productivity, and compromised welfare [27–29]. *Ascaridia galli* infections in poultry lead to substantial economic losses associated with treatment, prevention, and production. These losses encompass expenses for treatment, decreased productivity, and production losses for broilers, layers (including young and adult), and golden chickens [30]. *Heterakis gallinarum*, which is found in poultry caeca, not only causes pathological conditions in birds, but it also serves as a carrier of the fatal protozoan *Histomonas meleagridis* via infected eggs, resulting in the development of histomoniasis, a potentially fatal disease in poultry [3, 31]. Therefore, understanding the frequency, species variety, and geographic distribution of Ascaridids in poultry is critical for effective parasite management [3] elsewhere by considering varying susceptibilities and coinfections of metazoans and protozoans.

This scoping review is aimed at systematically mapping the global diversity, prevalence, and geographical distribution of poultry Ascaridid species reported worldwide up to 2026. In addition, a review on the prevalence of Ascaridids has specific continent and country‐wise among poultry birds under variable poultry rearing practices. Findings of this scoping review on the prevalence of Ascaridids will help prospective researchers working on poultry parasites, poultry industrialists, policymakers, and health care providers to poultry for prevention and treatment strategies for the well‐management of poultry worldwide. Mapping their distributions further supports proactive management, ensuring optimal poultry health, productivity, and the supply of safe products for consumers.

## 2. Material and Methods

### 2.1. Information Sources

**Figure 1:**
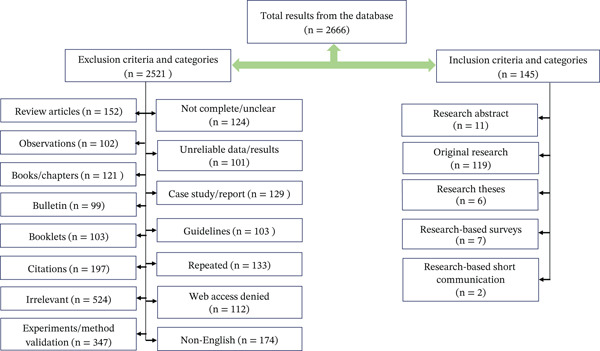
A flow chart showing the literature selection process and eligible articles for this review.

In this review, guidelines like PRISMA extension for reviews (PRISMA‐ScR) by Tricco et al. [32] were followed to increase the transparency in extracting research findings related to Ascaridida from published articles (*n* = 145) (original research [*n* = 119], theses [*n* = 6], research abstracts [*n* = 11], research‐based surveys [*n* = 7], and research‐based short communication [*n* = 2], Figure 1).

To ensure the quality and reliability of evidence and minimize bias, a comprehensive and systematic literature search was conducted across multiple electronic databases as stated above. Clearly defined inclusion and exclusion criteria were applied to select relevant studies. Study selection and data extraction were performed independently by more than one reviewer, and disagreements were resolved through discussion. Standardized data extraction forms were used to ensure consistency, and reference lists of selected articles were screened to identify further relevant studies, thereby reducing the risk of publication and selection bias.

### 2.2. Literature Searching Strategies and Selection of Source of Evidence

During the literature search, a total of 151 relevant literature (Figure 1) were identified using two common databases (Google Scholar [https://scholar.google.com] and PubMed [https://pubmed.ncbi.nlm.nih.gov]) and important search keys in different combinations (“Poultry, Nematodes,” “Poultry, Helminths,” “Poultry, Helminths, Parasites,” “Poultry, helminths, prevalence,” “Poultry, helminths, species,” “Poultry, helminths, distribution,” “Poultry, helminths, diversity,” “Poultry, *Ascaridia*,” “Poultry, *Heterakis*,” “*Ascaridia*, Species,” “*Heterakis*, Species,” “*Ascaridia galli*, Poultry,” “*Ascaridia galli*, Chicken,” “*Heterakis gallinarum*, Chicken,” “*Heterakis gallinarum*, Poultry,” “Poultry, Nematode,” “Poultry, Helminth,” “*Ascaridia*, Poultry,” “*Ascardia*, Infection,” “*Ascardia*, Infections,” “*Heterakis*, Infection,” “*Heterakis*, Infections,” “Helminth, Poultry,” “Helminths, Poultry,” “Infection, *Ascaridia*,” “Infections, *Ascaridia*,” “Poultry, Gastrointestinal parasites,” and “Chicken, Gastrointestinal parasites”). All articles searched for in each database were imported into EndNote 20 (Clarivate Analytics, Philadelphia, PA) to detect and remove duplicates and select eligible articles.

### 2.3. Eligibility (Inclusion and Exclusion) Criteria

The inclusive criteria encompassed comprehensive research or investigative articles focusing on the prevalence, distribution, and implications of poultry internal gastrointestinal helminth parasite species belonging to the Ascarididae family. Furthermore, research theses at the master’s or doctoral level that required clarity, detail, and cohesive results were evaluated if authorized by the relevant university. The requirements included research surveys, scientific communications, study notes with legitimate sampling procedures, extensive laboratory studies, and related results. Each part was classified using the inclusive selection criteria, ensuring a thorough examination of these parasitic organisms and their influence on poultry health and management techniques. Among 2666 results accessed, 145 literature (5.44%) met the criteria for original research having a thorough technique and precise data on parasite prevalence and distribution in poultry, and 2521 literature were declined due to exclusion criteria (Figure 1).

The exclusion criteria included a variety of materials and content kinds that were judged inappropriate for inclusion in the examination of chicken internal gastrointestinal helminth parasites within the Ascarididae family. This encompassed reviews, observations, advertisements, book chapters, bulletins, booklets, interviews, and citations. Additionally, articles considered irrelevant to the specified topic, experiments, or method validation studies, identification (coprological, morphological, microscopic, and molecular) of parasite species without prevalence rate, incomplete or unreliable (nonscientific) sources, case reports, or studies, guidelines, and content featuring repeated information were excluded from consideration. Furthermore, articles not in English were also part of the exclusion criteria, ensuring a focused and scientifically rigorous selection of materials for the investigation.

### 2.4. Data Management and Data Charting Process

Data were manually retrieved and placed into the Microsoft Excel sheet (Microsoft Excel for Microsoft 365 MSO Version 2311 Build 16.0.17029.20028). The retrieved information comprised authors, years, study areas of eligible studies, poultry and the size of their samples included in those studies, diagnostic procedures adopted by authors, and prevalence of respective species of Ascarididae. Tables, flow charts, and graphs were used to present our findings.

### 2.5. Data Analyses and Interpretations

Individual sources were critically appraised of evidence selected (Figure 1) and synthesized results. Further, the classification of Ascaridida was adopted by Taylor et al. [9] and analyzed species richness and diversity concerning the most recent comprehensive checklist of Ascrididae by Fedynich and Thomas [33]. Whenever there were multiple frequencies for bird types, rearing practices, prevalence rates of the particular parasite species, and geographical area, calculation was performed either the median or mean based on the normality of the data set for the specific variables. A meta‐analysis approach was adopted to interpret the prevalence of poultry Ascaridids. Global distribution of Ascarids infecting poultry (the details on continents, countries, and coordinates of the localities from where parasites were identified) was plotted in the global map using the software, Quantum Geographic Information System (QGIS, Version 2.13.3).

## 3. Results

### 3.1. Species of Ascaridids Infecting Poultry

**Figure 2:**
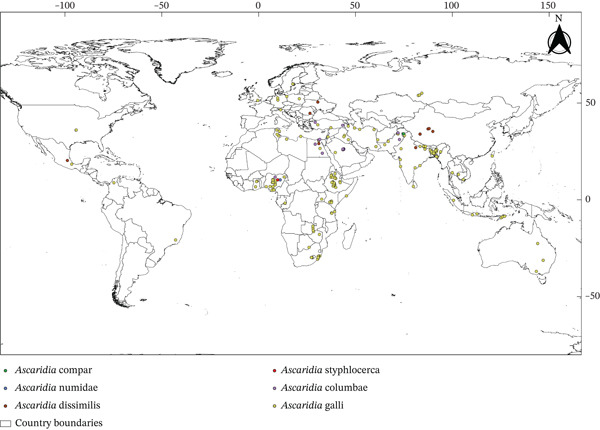
Global distribution of Ascaridia species infecting poultry (the details on continents, countries, and coordinates of the localities from where parasites were identified are available in Table S1).

**Figure 3:**
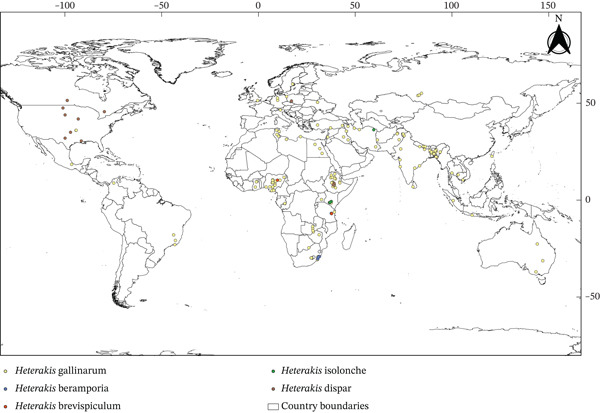
Global distribution of *Heterakis* species infecting poultry (the details on continents, countries, and coordinates of the localities from where parasites were identified are available in Table S1).

In this review, we found only two genera, that is, *Ascaridia* and *Heterakis*, representing 10 parasitic species belonging to the Ascarididae family (Table 1) known from among 151 eligible articles (Figure 1). The genus *Ascaridia* included six species (*Ascaridia columbae*, *Ascaridia compar*, *Ascaridia dissimilis*, *A. galli*, *Ascaridia numidae*, and *Ascaridia styphlocerca*) in the world (Figure 2). The *Heterakis* included five species (*Heterakis beramporia*, *Heterakis brevispiculum*, *Heterakis dispar*, *H. gallinarum*, and *Heterakis isolonche*) (Figure 3) in the world. These nematodes infected diverse species of poultry worldwide (chickens [*Gallus gallus* Linnaeus, 1758, *G. gallus domesticus* Linnaeus, 1758], ducks [*Anas platyrhynchos domesticus* Linnaeus, 1758, *Anas sparsa* Eyton, 1838] and Muscovy ducks [*Cairina moschata* Linnaeus, 1758], turkeys [*Meleagris gallopavo* Linnaeus, 1758, *Meleagris gallopavo silvestris*, Vigueras, 1931], pigeons [*Columba livia* Gmelin, 1789, *Columba livia domestica* Gmelin, 1789], pheasants [*Lophura leucomelanos* Latham, 1790], guinea fowls [*Numida meleagris* Linnaeus, 1758], domestic goose [*Anser anser domesticus* Kerr, 1792], and quails [*Coturnix coturnix* Linnaeus, 1758]; Table 1). The median sample size of these hosts included in this study was 148 (interquartile range [IQR]: 281–90).

**Table 1:** Checklist of Ascaridid nematodes infecting poultry and their prevalence worldwide (details in Table S1).

Species	Authority species	Host animal (species)	Continents (countries or regions)	Median prevalence (IQR)	References
*Ascaridia columbae*	Gmelin, 1790	Chickens (*Gallus gallus domesticus*)	Asia (Saudi Arabia, Iran, Pakistan)	12.09 (20.18–7.86)	[34–43]
			Europe (Turkey)		
		Pigeons (*Columba livia domestica*)	Africa (Giza Governorate and Aswan Province of Egypt, Ghana, Nigeria)		

*Ascaridia compar*	Shrank, 1790	*Gallus gallus domesticus*	Asia (India)	35.00 (NA)	[44]

*Ascaridia dissimilis*	Vigueras, 1931	Turkey (*Meleagris gallopavo*), turkey (*Meleagris gallopavo silvestris*)	**North America** (Kentucky, Tennessee, Georgia, Florida)	44.61 (63.13–25.25)	[45–53]
			Europe (Romania, Ukraine)		
			Africa (Egypt)		
			South America (Mexico)		

*Ascaridia galli*	Shrank, 1788	Chickens (*Gallus domesticus*, *Gallus gallus domesticus*), ducks (*Anas platyrhynchos domesticus*), turkeys (*Meleagris gallopavo*), ducks (*Anas sparsa*), pigeons (*Colombia livia*)	Africa (Botswana, Egypt, Ethiopia, Ghana, Gabon, Kenya, Lesotho, Libya, Nigeria, South Africa, Somalia, Tanzania, Tunisia, Uganda, Vietnam, Zambia, Zimbabwe)	32.25 (44.96–20.85)	[4, 6, 19, 21, 22, 35–37, 44, 54–128]
			Asia (Bangladesh, Cambodia, India, Indonesia, Iran, Iraq, Jordan, Nepal, Pakistan, Timor‐Leste, Sri Lanka, Taiwan, Thailand)		
			Australia, Europe (England, Germany, Italy, Poland, Sweden, Turkey)		
			North America (Arkansas)		
			South America (Mexico, Colombia)		

*Ascaridia numidae*	Leiper, 1908	Guinea fowls	Africa (Nigeria)	38.00 (NA)	[78]

*Ascaridia styphlocerca*	Stossich, 1904	Chicken	Africa (Nigeria)	4.50 (NA)	[129]

*Ascaridia* spp.	NA	Domestic goose (*Anser anser domesticus*), Muscovy duck (*Cairina moschata*), pigeons (*Columbia livia*), duck (*Anas platyrhynchos*), pheasants (*Lophura leucomelanos*)	Africa (Kenya, Nigeria)	19.60 (14.55–23.15)	[5, 20, 85, 112, 130–138]
			Asia (Bangladesh, Indonesia, Nepal, Saudi Arabia, Iraq)		
			Europe (Poland, Russia)		

*Heterakis beramporia*	Lane, 1914	Chicken	Africa (South Africa)	9.50 (NA)	[91]

*Heterakis brevispiculum*	Gendre, 1911	Chicken	Africa (Nigeria, Tanzania)	12.50 (9.75–15.25)	[86, 129]

*Heterakis dispar*	Shrank, 1790	Chicken, geese	Africa (Ethiopia, Tanzania)	31.50 (53.68–13.63)	[23, 77, 100, 139]
			North America (Texas)		
			Europe (Poland)		

*Heterakis gallinarum*	Shrank, 1788	Chicken, ducks (*Anas platyrhynchos domesticus*), ducks (*Anas sparsa*), guinea fowls, pigeons (*Colombia livia*), pigeons (*Colombia livia domestica*), turkey (*Meleagris gallopavo*)	Africa (Botswana, Egypt, Ethiopia, Gabon, Ghana, Kenya, Lesotho, Libya, Nigeria, South Africa, Tanzania, Tunisia, Uganda, Vietnam, Zambia, Zimbabwe)	19.62 (42.81–10.40)	[4, 6, 19, 21, 22, 35–37, 44, 53–119, 121–125, 127, 140–142]
			Asia (Bangladesh, Cambodia, India, Iran, Indonesia, Jordan, Nepal, Pakistan, Taiwan, Iraq, Sri Lanka),		
			Australia, Europe (Egypt, England, Germany, Italy, Poland, Sweden, Turkey, Ukraine)		
			North America> (Arkansas)		
			South America (Brazil, Colombia)		

*Heterakis isolonche*	Von Liston, 1906	Chicken, duck	Africa (Kenya, Tanzania)	9.00 (3.50–11.85)	[73, 86, 100, 113, 140]
			Asia (Iran)		

*Heterakis* spp.	NA	Chicken, domestic goose (*Anser anser domesticus*), duck (*Anas platyrhynchos*), pigeons (*Columbia livia*), guinea fowls (*Numida meleagris*), pheasant (*Lophura leucomelanos*)	Africa (Nigeria, Ghana, Zambia), Asia (Bangladesh, Indonesia, Nepal), South America (Mexico), Europe (Russia)	9.80 (2.20–18.30)	[5, 19, 68, 85, 95, 132–135, 137, 138]

*Note:* Range: maximum value minus minimum value in percentage.

Abbreviation: IQR, interquartile range, which equals to the third quartile (i.e., Q3) minus the first quartile (i.e., Q1).

### 3.2. Geographical Distribution and Prevalence of Poultry Ascaridids

Ascaridids have been reported in all continents (Africa [*n* = 62 studies], Asia [*n* = 52], Europe [*n* = 17], South America [*n* = 5], North America [*n* = 8], and Australia [*n* = 1]; Table S1) except in Antarctica and 40 countries (Table 1 and Table S1 and Figures 2 and 3). The overall median prevalence of Ascaridid species infecting studied hosts (Table 1) was 24% (IQR: 41.07–12%). *A. galli* and *H. gallinarum* were the cosmopolitan nematodes reported with median prevalences of 28.75% and 17.20%, respectively (Table 1 and Tables S1 and S2).

### 3.3. Prevalence of Ascaridids Based on the Poultry Bird Types

Eleven species of Ascaridids (Table 1) were known to infect eight different bird types (chickens, ducks, geese, guinea fowl, pheasants, pigeons, quails, and turkeys). The median sample size of chickens was 150 (IQR: 300–100), ducks 145 (IQR: 201–110), geese 21 (IQR: 24–18), guinea fowls 99 (IQR: 123.5–74.5), pigeons 120 (IQR: 240–60.75), and turkeys 175 (IQR: 525–56.5) whereas the studied pheasant and quail bird samples were 200 and 230, respectively. The prevalence of those nematodes is described in Table 2. These Ascaridid parasites mostly infected chickens (Table 2 and Table S3). The chickens were infected with nine species, ducks with three species, geese with two species, guinea fowls with two species, pheasants with two species, pigeons with three species, quail birds infected with a single species, and turkeys with three species of Ascaridids.

**Table 2:** Ascaridid nematodes infecting host bird types and the prevalence of their infection (details in Table S3).

Host types	Parasites	Prevalence percentage			References
		Median	IQR	Range	
Chickens	*A. numidae*	38	38–38	38–38	[4, 6, 19, 21, 22, 35–37, 44, 54–121, 123–125, 127–129, 132, 135, 137, 140–165]
	*A. compar*	35	35–35	35–35	
	*A. galli*	33.15	45.08–21.16	96.2–3.7	
	*H. gallinarum*	22.90	53.86–11.96	100–1.1	
	*H. brevispiculum*	12	12–12	12–12	
	*H. dispar*	16	44.86–11.25	73.71–6.5	
	*H. isolonche*	10.38	25.55–6.925	66.65–1	
	*H. beramporia*	9.5	9.5–9.5	9.5–9.5	
	*A. styphlocerca*	4.5	4.5–4.5	4.5–4.5	

Ducks	*A. galli*	14.44	28.94–12.5	36.8–0	[6, 74, 93, 113, 134, 138]
	*H. gallinarum*	10.1	15.15–4.3	28.4–0	
	*H. isolonche*	3.5	3.5–3.5	3.5–3.5	

Geese	*A. galli*	20	20–20	20–20	[134, 139]
	*H. dispar*	47	47–47	47–47	

Guinea fowl	*A. galli*	51.45	58.18–44.73	64.9–38	[95]
	*H. gallinarum*	11	15.5–6.5	20–2	

Pheasant	*A. galli*	11	11–11	11–11	[5]
	*H. gallinarum*	7	7–7	7–7	

Pigeons	*A. galli*	33.30	35.26–27.80	37.21–22.30	[20, 34–36, 39–41, 74, 119, 126, 130, 131, 134, 136, 166–168]
	*A. columbae*	12.09	20.18–7.86	83.3–1.4	
	*H. gallinarum*	8.84	16.23–3.95	18.7–0.9	

Quail	*A. galli*	52.17	52.17–52.17	52.17–52.17	[79]

Turkeys	*A. galli*	26	26.75–22.6	70–10.2	[53, 66, 93, 110, 117, 118, 169]
	*H. gallinarum*	12.90	22.63–10.20	70–1	
	*A. dissimilis*	44.61	63.13–25.25	94.8–6	[45–53]

*Note:* Range: maximum value minus minimum value.

Abbreviation: IQR, interquartile range, which equals to the third quartile (i.e., Q3) minus the first quartile (i.e., Q1).

### 3.4. Prevalence of Poultry Ascaridids for Different Management/Rearing Practices

The poultry birds were raised under three housing management systems. The following are the three major systems of rearing poultry birds: the free‐range or extensive poultry house system, the semi‐intensive poultry house system, and the intensive poultry house system. In addition, some of the poultry birds sampled for the Ascaridids investigation had unknown housing systems and were wild‐captured, and a few were migratory types (Table 3 and Table S3). The median sample size of eight poultry bird types reared under the extensive housing system was 167.5 (IQR: 290–100), under semi‐intensive housing was 100 (IQR: 163.75–44.5), under intensive housing was 150 (IQR: 373.5–70), and the unknown housing category was 140 (IQR: 200.75–100), whereas 230 birds were studied under the migratory system. Out of 11 poultry Ascaridids studied, birds under an extensive housing system were found to be infected with eight species, semi‐intensive with two species, intensive with two species, unknown housing with five species, and migratory housing systems with one species of nematode parasites (Table 3).

**Table 3:** Ascaridid nematodes infecting different poultry species and their prevalence based on different management practices (details in Table S3).

Management practices	Parasites	Prevalence percentage			References
	Species name	Median	IQR	Range	
Extensive	*A. galli*	34.25	45.90–20.61	96.2–0	[4, 6, 19–22, 31, 36, 37, 44, 54–57, 59–61, 63–118, 123, 127–129, 132, 135–137, 139–144, 158, 162, 165, 170]
	*A. compar*	35	35–35	35–35	
	*A. numidae*	38	38–38	38–38	
	*H. gallinarum*	30.35	59.72–15.33	100–0	
	*H. isolonche*	6.2	6.2–2.88	11.85–1	
	*H. dispar*	16	36.88–11.25	47–6.5	
	*H. beramporia*	9.5	9.5–9.5	9.5–9.5	
	*H. brevispiculum*	12	12–12	12–12	

Semi‐intensive	*A. galli*	26.75	57.79–19.05	71.5–12.9	[36, 45, 47, 49, 51, 58, 65, 81, 85, 118, 137, 171]
	*H. gallinarum*	15.62	20–6.94	72.3–0.6	
	*A. dissimilis*	39.22	53.9–26	72–25	

Intensive	*A. galli*	24	38–16.89	65.38–0	[21, 36, 41, 42, 53, 58, 63, 65, 83, 84, 89, 105, 108, 111, 114, 119, 120, 124–126, 137, 144–157, 161, 163, 164, 167–169, 172–176]
	*H. gallinarum*	13.53	18.46–4.93	59.33–0	
	*A. columbae*	10.02	14.98–7.86	20.7–1.4	
	*A. dissimilis*	4.14	5.31–2.97	6.48–1.8	

Migratory	*A. galli*	52.17	52.17–52.17	52.17–52.17	[79]

Wild‐captured	*A. dissimilis*	66.2	94.8–29.47	94.8–6	[48–50, 52]

Unknown	*A. galli*	21.67	36.10–14	64.9–0	[6, 34, 35, 38, 40, 43, 62, 71, 90, 95, 97, 98, 106, 112, 117, 121, 122, 129–135, 138, 140–142, 159, 177–179]
	*A. columbae*	13.3	52.55–7.70	83.3–5.83	
	*A. styphlocerca*	4.5	4.5–4.5	4.5–4.5	
	*H. gallinarum*	10.41	18.7–5.2	96–0	
	*H. isolonche*	66.65	66.65–66.65	66.65–66.65	

*Note:* Range: maximum value minus minimum value.

Abbreviation: IQR, interquartile range, which equals to the third quartile (i.e., Q3) minus the first quartile (i.e., Q1).

## 4. Discussion

### 4.1. Poultry Ascaridid Species Diversity and Geographical Distribution Patterns

In the present review, six *Ascaridia* species infecting poultry (Table 1) among 41 *Ascaridia* spp. [11] and five *Heterakis* spp. (Table 1) among a total of 29 *Heterakis* spp. [11] known to infect poultry as well as nonpoultry hosts (birds belonging to the wild, captive, and domestic collections) were confirmed [11, 180]. In this review, 11 species of Ascaridids were determined to belong to the genera *Heterakis* and *Ascaridia*, the most species‐rich genera in the Ascarididae family. Most studies (71.72%) were conducted during the last two decades, and the rest (28.28%) were conducted before 2000 (Table S1). Before 1990, the proper records (*n* = 9) regarding Ascaridids infecting poultry birds primarily documented *A. galli*, *H. gallinarum*, and *H. dispar*. Between 1990 and 2000, reports (*n* = 3) were well‐documented for poultry Ascaridids, including *A. galli* and *H. gallinarum*. Between 2000 and 2010, well‐documented reports (*n* = 11) were made about the prevalence of *A. galli*, *A. columbae*, and *H. gallinarum*. However, between 2010 and 2020, well‐documented reports (*n* = 54) for most of poultry Ascaridids included *A. galli*, *A*. *columbae*, *H. gallinarum*, and *H. isolonche*. The most number of documents (*n* = 74) regarding Ascaridids in the last 6 years include *A. columbae*, *A. dissimilis*, *A. galli*, and *H. gallinarum* (Table 1 and Tables S1 and S2). The list of host birds and their infecting Ascaridids was reported in the previous checklist, but lacked prevalence rates [11, 103]. In some systematic reviews and meta‐analyses, various classes of helminth parasites were included without emphasizing the Ascaridid species infecting poultry [103]. Thus, for the first time, comprehensive documentation of this review visualizes the Ascaridid species richness that infects poultry birds in a global scenario.

Poultry Ascaridids are intestinal nematodes distributed worldwide, with *Ascaridia* spp. being the most prevalent, followed by *Heteraki*s spp. which are the common nematode species infecting poultry birds [8, 134] also infected poultry (Table 1). This shows transmission pathways and a broad host range of Ascaridid infections among poultry and wild birds [180]. However, we also did not access a report describing poultry Ascaridids infesting human populations.

Most prevalence studies were conducted in Africa (*n* = 64), followed by Asia (*n* = 54), Europe (*n* = 19), South America (*n* = 5), North America (*n* = 8), and Australia (*n* = 1). The greater research interest and prevalence of poultry Ascaridids in Asian and African countries could be due to traditional free‐range poultry production systems in these regions [2]. Fedynich reported that *Heterakis* and *Ascaridia* species are geographically widespread, with at least one species from each genus found on every continent except Antarctica [11, 33]. The worldwide geographical patterns of poultry parasites have several advantages, including enhanced disease management and biosecurity by identifying high‐prevalence areas. In addition, improving surveillance of anthelmintic resistance in Ascaridid nematodes supports evidence‐based parasite control strategies and helps in the spread of drug‐resistant strains. This knowledge supports effective, region‐specific prophylaxis and treatments and efficient resource allocation and serves as a valuable resource for researchers. Policymakers can use this information to develop effective regulations and improve food security and the poultry industry. Moreover, analyzing parasite distribution trends offers early indications of outbreaks, boosts poultry health and productivity, and offers economic benefits to farmers and the industry.

### 4.2. Prevalence of Ascaridids Based on the Host Bird Types and Rearing Practices

The prevalence rate and diversity of Ascaridids among poultry were inconsistent (Table 1) with the global perspective because of various factors. The common factors are the different geography and host types, diagnostic techniques, dietary and health status of poultry, sources of parasitic infection, housing management practices, and poultry breeds [114, 129]. The inconsistent reports of species in the articles that we accessed (Figure 1) used various diagnostic methods, either conventional morphometry [57, 90, 93, 100] or advanced molecular [19–22] or both in combination [38, 79, 91]. The common sources of infection among poultry birds were reported to be migratory and nonmigratory wild birds at domestic interfaces, feral birds, improper disposal of slaughtered poultry materials, contaminated feeding materials, or even the possibility of vertical as well as horizontal modes of transmissions among the same species, breed of birds, or the different species of poultry birds [37, 65, 69, 107, 118, 136]. Therefore, *A. galli* is known to infect wild birds [8] and can potentially infect poultry where wild birds visit the operations without restriction. Integrated diagnostic approaches, with greater emphasis on modern molecular techniques, are therefore crucial for detecting species‐specific genotyping patterns associated with resistance to commonly used anthelmintics. In addition, elucidating parasite transmission dynamics and genetic alterations is for strengthening the biosecurity strategies.

A free‐ranging backyard scavenging system of raising poultry birds like chickens, ducks [113], hens, turkeys, pigeons [136, 177], and pheasants [37, 74, 93, 107, 136] was another factor responsible for the transmission of the extensive type of rearing management in most developing countries. This is because, in this rearing system, poultry birds can ingest the infected paratenic hosts (insects, flies, and earthworms) or contaminated feeding and drinking [37, 73, 107]. This indicates the possibility of variations in the prevalence rate of Ascaridids depending on the type of husbandry practices for poultry production on an individual farm and bird basis [84, 97, 101, 114]. Since these nematodes (Table 1) infected economically significant poultry birds such as chickens, pheasants, and turkeys, wild‐caught captive birds in zoological gardens, and pen‐raised game birds across almost all continents, excluding Antarctica [11], our checklist and analyses of prevalence can be used to formulate strategies to minimize the loss due to these Ascaridid’s infections, particularly for biosecurity measures.

Some poultry farmers collect and mix wild birds or their eggs with domesticated birds, or sometimes close contact at the wildlife interface may increase the risk of cross‐transmission of pathogens, including Ascaridids, from the reservoir to the naïve healthy bird [29, 95, 118]. Even in commercialized poultry production, poultry birds are reared under controlled environmental conditions, and there have been reports of infections from these Ascaridids species of parasites because of improper biosecurity and veterinary health care [89, 134, 181]. In most cases, there was the absence of trematode infections among poultry birds [54, 100, 129, 178], which could highlight either the lack of intermediate hosts like molluscs or that they are reared under the intensive management system of poultry production [20]. However, in the various agroclimatic conditions and differences in the seasons, the abundance of Ascaridid species among poultry birds varies [71, 96]. Similarly, the parasitic infection rate may fluctuate according to the type of breed, for example, between native/indigenous and exotic poultry breeds [135]. These findings emphasize the need for improved biosecurity to prevent contact between poultry and wild birds, regular veterinary monitoring, and targeted parasite surveillance. Variations in Ascaridid infections across seasons, agroclimatic conditions, and poultry breeds indicate that parasite control and deworming strategies should be tailored to local environments and breed susceptibility rather than applied uniformly, thereby improving sustainable poultry health management.

### 4.3. Constraints of the Study

Articles not accessible in the designated database could be available in another database. Thus, our report on the Ascaridid species checklist and their infection prevalence may have limitations requiring systematic reviews, including additional databases. Although incorporating diagnostic methods, seasonality, and regional differences could strengthen the analysis, the present study was limited to evaluating the prevalence, diversity, and geographical distribution of poultry Ascaridids.

### 4.4. Future Perspectives

All the previous research and review reports have mentioned the necessities of good husbandry practices in poultry farming, regular screening for parasitic infections, proper biosecurity and hygiene, awareness, and education among poultry farmers and entrepreneurs, along with proper anthelmintic use and veterinary health care. However, those reports failed to provide pinpoint guidelines to all the stakeholders. To provide clear guidelines, future research should not be focused only identifying helminths with molecular characterization but alon on their proper documentation especially on poultry Ascaridids. Research those focused only on identifying helminths followed by molecular characterization and documentation on poultry Ascaridids. There is still a need for a systematic review followed by research of different sources of parasitic infection in poultry birds and their mode of transmission and detection of parasitic life cycle checkpoints via an integrated approach involving governmental interventions and support from researchers, policymakers, and all the concerned stakeholders to promote commercialization and advancement of the poultry industry [182]. In addition, this review can be an avenue for the parasitic trait‐based evolutionary research regarding this arena. The systematic review and meta‐analyses could provide transparency in the infection rates, ideas about the most common species of parasites, their host specificity, and other major factors (climatic, geographical, and farming patterns). In addition, the increasing burden of anthelmintic resistance mechanisms should be monitored to mitigate the global problem in poultry production [180, 183, 184]. Moreover, research focusing on host gut microbiota modulation could provide novel strategies to mitigate parasitic burden in the poultry gastrointestinal tract [166, 185].

## 5. Conclusions

This scoping review concludes that poultry nematodes, particularly Ascaridid species, are widely distributed globally and pose a significant burden to poultry health. A total of 11 nematode species were identified across eight host types, with chickens being the most affected host, showing a median prevalence of 24% (IQR: 41.07%–12%). Globally, *A. galli* and *H. gallinarum* were the most common poultry nematodes reported, having median prevalences of 28.75% and 17.20%, respectively. Six *Ascaridia* and five *Heterakis* species were reported in chickens worldwide, with higher prevalence observed in Africa and Asia, likely reflecting extensive and free‐range rearing practices. The cosmopolitan distribution of these nematodes and their potential transmission at the wildlife‐poultry interface emphasize the broad exposure risk. Overall, the occurrence and diversity of Ascaridid infections vary according to geographical region, host type, and rearing systems, underscoring the importance of region‐ and system‐specific parasitic management strategies. Our checklist of these parasites can be helpful in prioritization strategies and actions for developing prevention and control of poultry Ascaridids and planning more sophisticated research in the field of therapeutics for mitigating the problem of anthelmintic resistance, management, and health–welfare to lower the infection rates in poultry for specific countries worldwide.

## Author Contributions

C.R.P. prepared the original draft, conceptualized, analyzed and interpreted data, reviewed and edited the manuscript, and provided validation. D.P.P. edited and reviewed the manuscript and supervised. P.K. edited and reviewed the manuscript and supervised.

## Funding

No funding was received for this manuscript.

## Disclosure

All authors read and approved the final manuscript.

## Ethics Statement

The authors have nothing to report.

## Consent

The authors have nothing to report.

## Conflicts of Interest

The authors declare no conflicts of interest.

## Supporting Information

Additional supporting information can be found online in the Supporting Information section.

## Supporting information


**Supporting Information 1** Table S1: Members of the Ascarididae family (nematodes) infecting poultry worldwide, their hosts, and prevalence including countries where these nematodes were originally reported.

**Supporting Information 2** Table S2: Global distribution of Ascarids infecting poultry (the details on continents, countries, and coordinates of the localities) from where parasites were identified.

**Supporting Information 3** Table S3: Prevalence of Ascaridids’ infections with respect to hosts (i.e., poultry species) and rearing practices.

## Data Availability

The data that support the findings of this study are available from the corresponding authors upon reasonable request.
